# Combating climate change through sustainable cattle ranching in the global south: The role of societal corporatism

**DOI:** 10.1007/s13280-024-02051-2

**Published:** 2024-08-07

**Authors:** Thomas K. Rudel

**Affiliations:** Rutgers University, Emeritus, 53 Concord Avenue, Metuchen, NJ 08840 USA

**Keywords:** Climate change, Colombia, Corporatism, Ecuador, Politics, Silvopastures

## Abstract

The contours of the collective action necessary to limit climate change remain difficult to discern. In this context, societal corporatist political processes, fueled by crisis narratives, have shown some promise as political devices for mobilizing people. Corporatist processes have, historically, brought political competitors like employers and labor unions together to negotiate compacts that have advanced collective goods during times of crisis. In response to the climate crisis in the Global South, affluent donor groups, state officials, some farmers, and indigenous peoples have begun to assemble corporatist-like coalitions to pursue climate stabilization. A comparative case study of efforts to promote sustainable cattle ranching through the spread of silvopastoral landscapes in Colombia and Ecuador illustrates this political dynamic, its shortcomings, and its accomplishments.

## Introduction

Limiting climate change requires global scale collective action. Despite myriad research efforts by multitudes of social and natural scientists, we do not really know how to mobilize people to protect the environment at a global scale. Questions about ways to preserve large-scale public goods like a stable climate remain unanswered (Stern 2011). Some researchers have observed that the preservation of a large-scale commons only occurs if its plight achieves a high degree of salience among users (Yoder et al. 2022). Calamitous events and associated crisis narratives shock officials and citizens into sustained collective action (Rudel 2019). Under these circumstances, mobilization to preserve the commons occurs across a wide variety of strata in society, and the new regulatory regime promises to conserve the commons over extended periods of time (Ostrom 1990, 2010; Yoder et al. 2022).

These thoughts about workable schemes for collective action begin to flesh out what effective collective action would look like in the global arena, but they do not draw on the historical record of large-scale collective actions, oftentimes by nations, during the twentieth century. Many of these collective actions occurred at societal scales during wars. Several observers have picked up this analytic thread and argued that societies must mobilize as they did during wars in order to prevent the continued accumulation of greenhouse gases in the atmosphere (McKibben 2016; Klein 2020). These arguments about the need to approximate wartime mobilizations have been criticized for their vague or inaccurate depictions of wartime conditions (Wang and Smith 2020). There are, however, more precise arguments about wartime mobilizations that may shed some light on ways to mobilize large aggregates of people to reduce concentrations of greenhouse gases in the atmosphere. In particular, corporatism, a political process used frequently by nations during wartime, may hold out some promise as a way to generate large-scale collective actions to counter climate change.

This paper explains why a loose form of corporatist political processes could be expected to generate large-scale collective action to reduce climate change in the Global South. It begins by defining corporatism. Then, it explains in theoretical terms how crises and associated crisis narratives have provided the political impetus behind the creation of corporatist organizations, ‘committees of the whole,’ that mount collective efforts to secure public goods. The paper goes on to assess the connection between corporatism and large-scale collective action to promote sustainability. It does so through case studies of the conversion of pastures into silvopastures in cattle ranching regions of Colombia and Ecuador.

## What is corporatism?

During the Great Acceleration in the scale of human activities after 1945 (Steffen et al. 2015; McNeill 2016), observers frequently characterized societies as a mass of market participants regulated by the state. This depiction of societies omitted intermediary groups, like religious congregations or economic sectors (Durkheim 1897). These intermediary groupings take on a corporate quality when participants unite in pursuit of a particular goal (Wade 1988). When historical circumstances strengthen intermediary groups, corporatist political processes become prevalent. In the best known of these processes, participants in an economic sector come together, workers in a sector as well as the owners of enterprises in a sector. These different actors negotiate a series of compacts, agreements in which the different interest groups become parties to political exchanges. For example, facing the dire circumstances of a recession, participants in the industrial sector of an economy, at the behest of government regulators, agree to a trade-off. Employers promise not to lay off workers if labor unions promise to limit their wage demands. In this instance, a unified group in an economic sector arrived at an agreement among themselves that achieved a collective goal of limiting the economic pain from a recession or some other kind of crisis. This is corporatism. Of pertinence to inquiries about combatting climate change, corporatist negotiators often represent an entire sector of an economy, so their compacts shape large-scale economic aggregates and have the potential to reform large-scale commons.

**Fig. 1:**
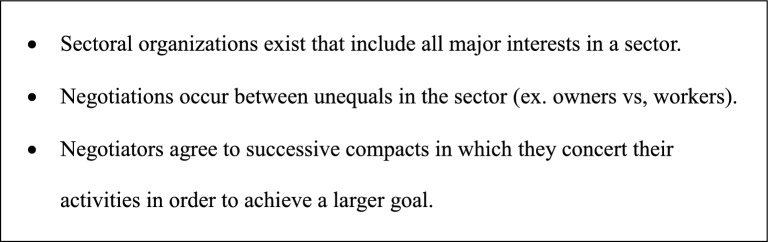
The distinguishing characteristics of corporatist political processes

Figure 1 lists the distinguishing characteristics of corporatist polities. The bedrocks of corporatist polities are sectoral organizations whose limits are defined by a focal activity like manufacturing. These sectoral organizations vary in scale. They can incorporate an entire national economy, as in examples of corporatism from World War I (McKillen 1991). Alternatively, they can involve relatively small numbers of indigenous people, public servants, and foreign conservation advocates. In the example of a corporatist polity presented below, the sector includes cattle ranchers, government extension agents, and NGO personnel who manage a flow of payments for environmental services.

Several other characteristics of corporatist political processes stand out. First, the negotiations within sectors often occur between unequals. Representatives from the working class negotiate with employers and government officials who are drawn from the elite strata of societies. Second, the compacts in corporatist political processes recur over time. The negotiators meet periodically to review the performance of their policies during the preceding period, and, on the basis of these performances, they draw up a new compact for a subsequent period. A year after a first compact, the same parties meet and negotiate an updated agreement that reflects the somewhat altered circumstances of the new year.

**Fig. 2:**
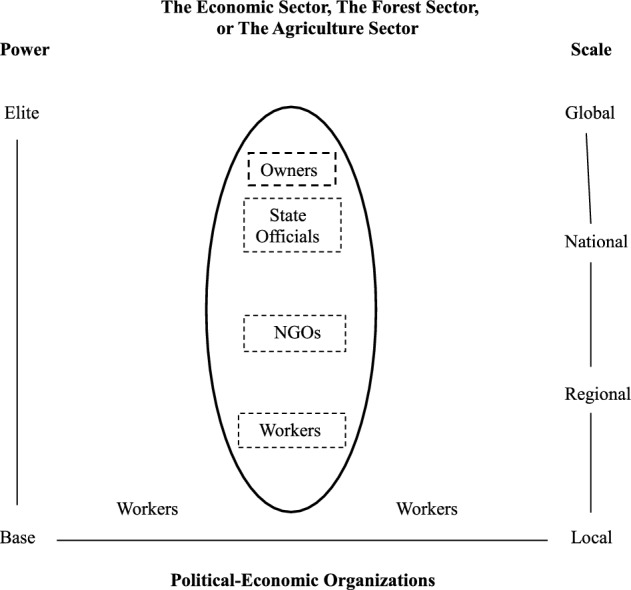
Political configurations in a corporatist polity

**Fig. 3:**
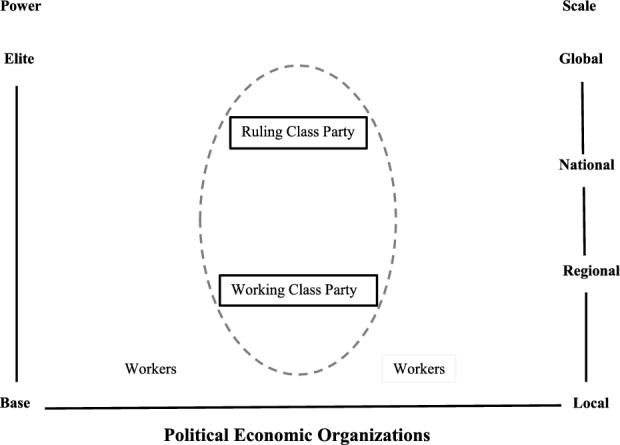
Political configurations in a pluralist polity

A comparison with a different type of polity may clarify the distinguishing characteristics of corporatist political processes. The diagrams in Figs. 2 and 3 map out the configurations of corporatist (Fig. 2) and pluralist (Fig. 3) political orders. Political dimensions define the organizational maps. Power stratifies from impoverished groups, the base, and well-endowed groups, the elites. The scale of an organization’s activities varies from local to global undertakings.

The organizational maps in Figs. 2 and 3 also distinguish between more and less autonomous organizations. The more autonomous and powerful organizations have solid line perimeters. Organizations with dash-lined perimeters have less power. They typically follow plans put forth by organizations with solid line premeters.

The corporatist political configuration in Fig. 2 brings together diverse groups within a single, sector-wide organization to address a crisis. Variously referred to as ‘committees of the whole,’ ‘encompassing organizations,’ or ‘sectoral associations,’ these organizations create the plans that guide activities in corporatist polities. Politicians create this kind of organization when the crisis framing of an issue like climate change becomes undeniable to influential actors and, out of alarm, they take action to address the issue. The oval in Fig. 2 depicts this crisis-generated, politically strong sectoral organization. As implied by this organizational map, most of the political contention in a corporatist polity occurs within the confines of a sector’s encompassing organization.

Alarm also spreads in a pluralist polity when the crisis framing of an issue becomes undeniable, but the post-alarm, political organizational map looks different. As displayed in Fig. 3, the alarmed politicians gather in groups with different material interests who contend with one another for control of the polity (Dahl 1978). This political order has few encompassing organizations (Olson 1982). For this reason, pluralist political orders are less likely than corporatist political orders to arrive at agreements that enable them to undertake large-scale collective actions. To denote the weaker capabilities of sectoral organizations in pluralist political orders, they are denoted with dashed boundaries in Fig. 3.

The history of corporatist political processes in twentieth century Europe provides a measure of the potential of corporatist political processes for mobilizing people around the goal of climate stabilization. At the outset of World War I, many of the combatants adopted corporatist processes in order to increase the likelihood that industrial and labor leaders would comply with the extraordinary measures required by a society’s mobilization for war (McKillen 1991, 2010). In the interwar period, fascist regimes in Spain, Italy, and Germany adopted corporatist processes, in part as in the World War I cases, to extract more production out of people in the industrial sector. ‘State corporatism’ best describes these early twentieth century regimes because state officials exercised a very large degree of control over the substance of the compacts between employers and workers (Schmitter 1974).

After World War II, the Scandinavian countries, West Germany, and Austria adopted a kind of ‘societal corporatism’ in which democratically constituted labor unions negotiated on relatively even terms with employers and government officials (Schmitter 1974). Reviews of these postwar compacts have concluded that societal corporatist regimes did particularly well, compared to pluralist polities, in policy responses to periodic economic crises (Molina and Rhodes 2002; Gronow et al. 2019).

In these historical episodes, crises prepared the ground for corporatist processes because crisis narratives provided the impetus for the creation of the sectoral organizations that mobilized people. Credible crisis narratives helped sector participants overcome any hesitation that they may have felt about entering into compacts across class lines. A similar dynamic might explain the emergence of corporatist processes in response to the crises posed by climate change. In sum, corporatist collective action tends to occur in response to crises, engages activists in sectoral organizations that cross class lines, and occurs at a variety of scales. Taken together, these attributes suggest that corporatist processes could play an important role in climate change mitigation.

Several aspects of this historical record raise questions about this optimistic assessment of corporatism as a political device for combating climate change. First, corporatism as a political process has waxed and waned in its prevalence, with diminishing application in some Scandinavian countries over the past two decades (Öberg et al. 2011). Reduced numbers of organizations that mobilized people would make it difficult to build the pan-organizational sectoral organizations that make corporatist processes work. Second, the historical experience of corporatism seems largely confined to industrialized, twentieth century European societies. Has a looser form of corporatist processes come to characterize efforts to combat climate change in the Global South? Case studies of corporatism distant in time and place from European settings would make it possible to assess the utility of corporatist processes for mobilizing efforts to mitigate climate change in diverse locales. To this end, the following case studies from Colombia and Ecuador should demonstrate the potential utility of corporatist political processes for advancing efforts to create more sustainable cattle ranching through the spread of silvopastoral land uses. The data for the case studies comes from two primary sources: national and international archives which describe the history of the Colombian project and interviews combined with fieldwork on 98 cattle ranches in a cattle ranching region of the Ecuadorian Amazon (Lerner et al. 2014).

## Did societal corporatism drive the spread of silvopastoral land uses in Colombia and Ecuador?

### The Latin American context

The case studies begin with a brief description of the coupled natural and social context in which silvopastures spread in Ecuador and Colombia. Like other nations in Latin America, both Colombia and Ecuador contain extensive natural grasslands. Early Spanish settlers brought a devotion to cattle ranching to the Americas, and their enthusiasm for it fostered the spread of cattle ranching throughout the grasslands of Latin America during the seventeenth and eighteenth centuries (Shane 1986; Van Ausdal and Wilcox 2013). This colonial legacy has persisted into the twentieth and twenty-first centuries. Thirty percent of the global emissions from the cattle sector currently occur in Latin America (Solymosi et al. 2016; Lerma et al. 2022). Colombia in particular has made substantial contributions to this continental pattern. It was the seventeenth largest producer of cattle in the world in 2015 (USAID 2015).

Small producers predominate in the Colombian and Ecuadorian cattle sectors. Forty-seven percent of the cattle ranchers in Colombia had fewer than ten head of cattle on their farms. Two-thirds of the cattle ranchers in Ecuador work less than 20 hectares of land (Barahona and Baillard 2015; USAID 2015). These small-scale ranchers have suffered from other challenging conditions. Rural areas contain few farm to market roads, and they are frequently impassable, which disadvantages smallholders when they try to market their cows. Most smallholders do not have secure titles to land which restricts their access to credit from banks (USAID 2015). The lack of access to loans in turn makes it difficult for smallholders to finance the conversion of pastures into silvopastures.

Until very recently, Latin American landowners raised cattle in extensive systems of open fields. Both Ecuador and Colombia had on average less than one cow per hectare of pasture (Barahona and Baillard 2015; USAID 2015). The extensive cattle ranching in both countries has historically fueled a ruinous process of environmental destruction through the conversion of old growth forests into pastures (Shane 1986). Among small, poorly resourced ranchers in these districts, without funds to renew soil resources with fertilizers, a *barbecho* crisis has emerged in which the growth of pasture grasses has slowed and their capacity to sustain growth in cattle has declined (Maxwell 1980; Thiele 1993; Bates 2007). Under these circumstances, smallholders began to look for alternative sources of income from their land.

At the same time, persistently high rates of tropical deforestation and associated surges in greenhouse gas emissions alarmed leaders in international conservation organizations and sympathetic governments (Rudel 2023). A crisis narrative fueled by rural poverty, threats to tropical biodiversity, and climate change began to reshape political and economic agendas across a wide range of Latin Americans from international leaders to environmental activists and small-scale ranchers. In this context of economic distress and growing concern about environmental destruction, silvopastures have become an attractive land use option for ranchers. In Colombia, as described below, a corporatist pattern of governance has emerged to drive the expansion of silvopastures.

### Silvopastures and societal corporatism in Colombia

Cattle ranching in Colombia occurs in a wide range of climates, some very humid like the eastern slope of the Andes in southeastern Colombia and others with limited rainfall like the inter-Andean valleys and portions of the eastern plains. In the more extensive and arid cattle ranching zones in Colombia, landholders who want to create silvopastures must purchase seedlings and pay to have them planted. These practices raise the cost of implanting silvopastures in Colombia to as much as $US4000 per hectare (USAID 2015).

Faced with declining levels of agricultural production and associated environmental damages from the conversion of tropical rain forests into pastures, diverse groups began to advocate for sustainable intensification in the livestock sector in which incomes from pastures would increase at the same time that environmental conditions in and around pastures would improve. The conversion of pastures into silvopastures would promote both of these ends. The addition of trees to pastures would provide farmers with an additional stream of income from the sale of trees, sequester some carbon, and foster the recovery of plant and avian biodiversity in and around the emerging trees. In recent studies, planted silvopastures in Colombia and spontaneous silvopastures in Ecuador both sequestered about 1.5 tons of carbon per hectare per year (McGroddy et al. 2015; IUCN 2022). Given these projected changes, people interested in promoting a more sustainable cattle sector began to express an interest in fostering the conversion of pastures into silvopastures.

International conservation NGOs, bilateral aid officials from Europe, climate finance bankers, companies selling sustainable dairy products, public officials concerned with IPCC commitments, extension agents for cattle ranchers, and small-scale cattle ranchers all expressed an interest in accelerating the conversion of pastures into silvopastures in Colombia. Beginning shortly after 2000, the interested parties put together annual or biennial roundtable gatherings in which participants described, discussed, and made commitments to promote silvopastoral landscapes. A network of roundtables emerged. An international roundtable met periodically. A national roundtable, and twelve provincial roundtables began to meet in Colombia (Lerma et al. 2022). These sector specific gatherings coordinated and concerted the activities of the roundtable participants, much the way that the sector-wide organizations did in the corporatist polities of twentieth century Europe.

For their part, government officials in Colombia began to work on a national plan for the livestock sector that reflected an emphasis on sustainable intensification. In Colombia’s National Development Plan, pastures would decline by 10 million hectares, from 38 to 28 million hectares, while the cattle herd would grow from 23 to 40 million head. In accordance with the national plan, government officials formulated a National Public Policy for Sustainable Cattle that prioritized the conversion of pastures into silvopastures (USAID 2015).

The high costs of purchasing and planting valuable species of trees, coupled with the continuing challenges of managing the growth of the newly planted trees, created pressing needs among farmers for revenue to finance the conversion of pastures to silvopastures. At the same time, the enthusiasm for the Colombian plan for sustainable cattle ranching among international participants in the roundtables led to financial commitments to pay for the environmental services generated by the emerging silvopastures. The Global Environmental Facility (GEF) and the United Kingdom’s Department of Energy and Climate pledged 8 million dollars and 15 million pounds, respectively, to pay for the carbon sequestered in the pilot project’s emerging silvopastures. Summed from all of the various sources, national and international donors contributed $US42 million dollars to the sustainable cattle project between 2008 and 2016. These funds paid for environmental services, and, in so doing, defrayed some of the smallholders’ expenses in establishing the silvopastures.

Smallholder cattle ranchers with herds of less than fifty animals could not afford the $US 4000 per hectare costs of converting from pastures to silvopastures. A majority of these smallholders needed subsidized credit to make the change. To meet this need, public sector bankers at FINAGRO (*Fondo para el Financiamiento del Sector Agropecuario*) created a program of subsidized credit for smallholders who wanted to convert pastures into silvopastures (USAID 2015).

To facilitate the spread of silvopastures in the second phase of the project, the government created 40 experimental farms with silvopastoral demonstration plots across all of the major cattle ranching regions in Colombia (USAID 2015). Extension agents, small-scale ranchers, and personnel from environmental NGOs participated in a series of workshops at these farms on the advantages of silvopastoral land uses (IUCN 2022). Deep pocketed dairy companies who marketed their products to the urban populations of Colombia also promoted silvopastures, but their efforts were focused on the larger farms that supplied the companies with milk. Company executives pursued this sustainable cattle emphasis despite little demand from Colombian consumers for sustainable dairy products. Relatively little coordination has occurred between the companies in their sustainability efforts. Environmental NGOs like the Nature Conservancy and the Center for Research on Sustainable Agricultural Production Systems (CIPAV) did join working groups that have begun to focus on the restoration of forests on degraded pasture lands (USAID 2015).

The array of participants in Colombia’s silvopastoral program ranged across scales, as it usually does in corporatist processes, from local to national and global interests, but it changed over time with the deliberations of the roundtables. The following paragraphs outline these changes in the historical trajectory of the Colombian silvopastoral project.

The initial impetus for the sustainable cattle efforts began just after the millennium when rates of tropical deforestation reached very high levels throughout Latin America, and crisis narratives about deforestation and climate change proliferated. Political leaders, most notably Fernando Enrique Cardoso in Brazil, imposed new restrictions on land use on the Amazon basin, restricting land clearing to 20% of a claimed tract of forested land (Rudel 2023). In this context, the idea of promoting ‘sustainable cattle’ through payments for environmental services (PES) in emerging silvopastures first took root. Colombian government officials and representatives from the GEF and the UK began discussing a joint project, and the officials convened their first roundtables.

In the project’s initial term, smallholder cattle ranchers received PES payments for the conversion to silvopastures. Avian biodiversity increased in the converted pastures, and 90% of the participating farmers from the first term voted to participate in the project’s second stage. These markers of success persuaded the groups attending the roundtables that the silvopastoral project should be both extended and expanded. In its second term, beginning in 2012, the project area expanded to cattle ranching regions throughout Colombia with commensurate increases in the numbers of workshops and experimental silvopastoral farms across the regions (IUCN 2022). *Fedegan*, the national association of Colombian cattle ranchers, endorsed the second term project and joined the roundtables (Lerma et al. 2022). From the first to the second terms of the silvopastoral project, it gained momentum. To be sure, silvopastoral landscapes still have a long way to go before they become a dominant feature in the Colombian landscape. While three million hectares currently have silvopastures, as much as twenty million hectares of pasture remain in degraded conditions (USAID 2015).

**Fig. 4:**
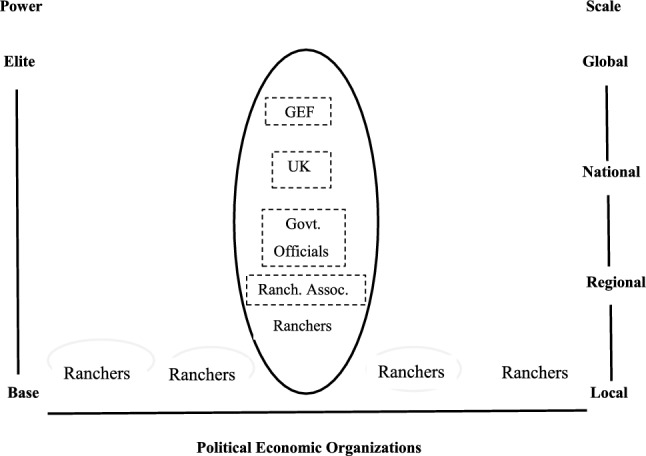
Political configurations in the spread of Colombian silvopastures

The sustainable cattle project in Colombia has not, to date, had an export dimension to it. Certification schemes (Sikor et al. 2013) have yet to emerge in which regulators permit beef raised in silvopastures to be sold in high-priced European markets. Still, the organizational response to the environmental crisis, the wide range of participating organizations ranging from international agencies to impoverished smallholders, and the emergence of roundtables that negotiate recurrent compacts that coordinate silvopastoral initiatives, taken together, resemble the corporatist dynamics described at the outset of this article. Figure 4 depicts the organizational shape of the sustainable cattle sector in Colombia.

### Spontaneous silvopastures and household efforts at sustainable cattle ranching in Ecuador

**Fig. 5:**
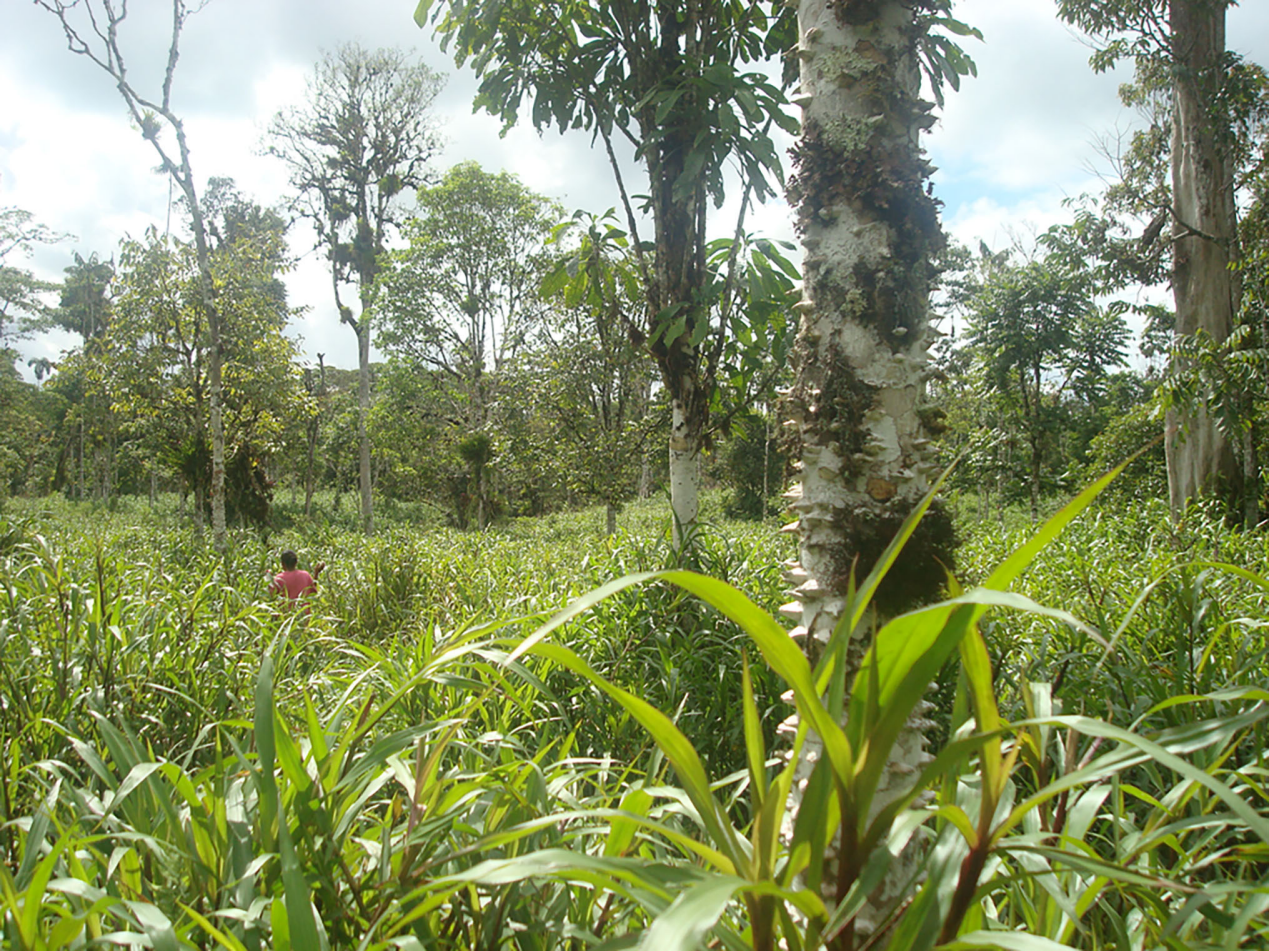
A spontaneous silvopasture in Ecuador. Photo Credit: Thomas K. Rudel

In Ecuador, variations in climate have influenced efforts to propagate silvopastoral systems. In recently deforested, wetter regions of the Ecuadorian Amazon with remnant patches of forest, tree seedlings germinate spontaneously in pastures. When smallholders in these places go out to the pastures to ‘change the cows’ to fields with mature grasses, they clear the just grazed pasture of tree seedlings which have germinated in the pastures. Left in place, these seedlings and saplings would begin to shade out the pasture grasses in several years. If the sprouting tree seedling is of a commercially valuable species, the smallholder will leave it in place to grow. Fifteen years later, the once spared tree may be large enough to sell to a sawmill. During the preceding decade, a silvopastoral field will have emerged spontaneously. Figure 5 depicts a spontaneous silvopasture north of Macas in the Ecuadorian Amazon. Worth noting here. The costs of establishing these silvopastures are minimal. The plants and planting cost nothing, given that the seedlings and saplings emerge spontaneously. Because the dominant pasture grass in the region, *gramalote* (*Axonopus scoparius*), is shade tolerant, the silvopastures maintain a stocking rate for cattle equivalent to nearby sun filled pastures. There does have to be a market for the sawmill’s wood, which implies that these spontaneous silvopastures will only emerge near cities.

With the minimal costs and palpable gains from establishing silvopastures in these districts, landowning smallholders began to allow trees to sprout. Surveys of fields across three communities indicated stem densities in excess of 200 trees per hectare of pasture in 2012 (Lerner et al. 2014). The emergence of silvopastures in these settings occurred as a smallholder initiative. Word of mouth and observations of trees sprouting in the pastures of neighboring farms fueled the conversion of pastures into silvopastures in landowning, smallholder cattle districts. At least in this setting, a reliance on observation and word of mouth to drive the spread of silvopastoral landscapes suggests that these land use changes will occur relatively slowly compared with places like Colombia where political decisions led to sudden expansions in silvopastures.

The state and international NGOs played minimal roles in the Ecuadorian land use changes. The profile of public assistance to small cattle ranchers takes a familiar form in this region. A state funded agrarian bank provides landowners with credit at concessionary rates. A government funded experiment station promotes favored crops like oil palm. Agricultural extension agents, with minimal budgets aside from salaries, make only occasional visits to smallholder cattle communities. The agendas of the central and provincial governments have focused primarily on road building.

In sum, silvopastures represented a ‘do it yourself’ project by smallholder Ecuadorian cattle ranchers. It almost goes without saying that this profile of sustainable development has not been driven by a corporatist political process. This negative case underscores how corporatism seems unlikely to occur in societies with low organizational densities like this district in the Ecuadorian Amazon. These societies lack the intermediary groups that bring different segments of society together in corporatist political processes. To the extent to which this organizational profile characterize rural districts in other countries in the Global South, it would be reasonable to conclude that these places would not generate corporatist political processes, even in circumstances characterized by salient crisis narratives.

## Conclusion

The two cases presented above outline both the limits and the potential accomplishments of corporatist political processes when applied to the governance of climate change in predominantly agricultural settings. The Ecuadorian case suggests some potentially insurmountable difficulties in constructing corporatist political processes. The organizational requisites for the institutionalization of corporatist political processes appear unlikely to occur in many impoverished societies with underfunded organizations run by leaders reluctant to undertake new initiatives. The Colombian example presents a brighter picture. International organizations and government officials took an interest in silvopastures that increased over time. The recurrent roundtables provided a venue where the interested parties, ranging from small farmers to international donors, could concert their efforts to promote silvopastoral expansion over time. The expansion in the extent of enrolled lands from the first to the second phase of the project attests to the potential for scaling up the spread of silvopastoral systems to the point where they could have global significance. The ability of this program to expand in the face of the high costs of planting trees attests to the strength of the driving forces unleashed by the creation of a corporatist governing group.

Granted, the participants in the silvopastoral governance group in Colombia do not replicate the array of interested parties in northern European corporatism. There is no tripartite structure of business owners, labor unions, and government officials that seems to be a constant feature in governance through corporatist processes in northern Europe. Still, the Colombian case does exhibit encompassing organizations, negotiations between unequals, and recurrent compacts that concert sustainability initiatives. In this looser form, corporatist political processes would seem to present a useful model of governance that could get things done in combatting climate change.

There are, however, important questions that these two case studies leave unanswered. First, an effective corporatist process would entail meaningful political exchanges between negotiators with substantial differences in political power. Neither one of the case studies contain evidence about these types of exchanges. Further work on corporatism in impoverished agricultural contexts should attend to this issue.

Second, the political effects of shocking events and crisis narratives in mobilizing encompassing organizations remain unclear. Clearly, the sharpening sense of a climate crisis played a role in mobilizing international organizations to participate in the Colombian silvopastoral project. The relative absence of international environmental NGOs on the eastern slope of the Andes in southern Ecuador may explain why the emerging crisis narrative about forest destruction in Latin America had so little impact on efforts to spread silvopastoral landscapes in this region.

Further case studies focused on the association between well-populated organizational fields, crisis narratives, and the emergence of corporatist political processes would verify whether or not corporatist processes have their origins in crisis narratives that become prevalent in environmentally concerned communities. If so, the construction of a corporatist political process may depend on the strength of the international social movement for climate stabilization in the surrounding society (Fisher et al. 2023). In any event, the research presented here demonstrates in one case a link between corporatist political processes and one small-scale solution to the climate—biodiversity crisis (Rudel 2023).
